# Fatty Acid Synthase Correlates With Prognosis-Related Abdominal Adipose Distribution and Metabolic Disorders of Clear Cell Renal Cell Carcinoma

**DOI:** 10.3389/fmolb.2020.610229

**Published:** 2021-01-25

**Authors:** Wenhao Xu, Xiaoxin Hu, Aihetaimujiang Anwaier, Jun Wang, Wangrui Liu, Xi Tian, Wenkai Zhu, Chunguang Ma, Fangning Wan, Guohai Shi, Yuan-Yuan Qu, Hailiang Zhang, Dingwei Ye

**Affiliations:** ^1^Department of Urology, Fudan University Shanghai Cancer Center, Shanghai, China; ^2^Department of Oncology, Fudan University Shanghai Medical College, Shanghai, China; ^3^Department of Radiology, Fudan University Shanghai Cancer Center, Shanghai, China; ^4^Department of Urology, Sun Yat-sen University Cancer Center, Guangzhou, China; ^5^State Key Laboratory of Oncology in Southern China, Guangzhou, China; ^6^Department of Neurosurgery, Shanghai East Hospital, Tongji University School of Medicine, Shanghai, China

**Keywords:** FASN, visceral adipose tissue, clear cell renal cell carcinoma, prognosis, metabolism, obesity

## Abstract

**Purpose:** Lipid metabolism reprogramming is a major pathway in tumor evolution. This study investigated fatty acid synthase (FASN) mRNA expression in anthropometric adipose tissue and elucidated the prognostic value and potential mechanism of clear cell renal cell carcinoma (ccRCC).

**Materials and Methods:** Transcription profiles were obtained from 533 ccRCC samples in The Cancer Genome Atlas (TCGA) cohorts. Real-time quantitative PCR (RT-qPCR) and immunohistochemistry were performed to detect *FASN* expression in 380 paired ccRCC and normal tissues from the Fudan University Shanghai Cancer Center (FUSCC). Visceral adipose tissue (VAT) and subcutaneous adipose tissue were at the level of the umbilicus as measured by magnetic resonance imaging (MRI). Non-targeted metabolomics and *in vitro* experiments were used to reveal the biological functions of FASN.

**Results:** Increased *FASN* expression was significantly relevant to advanced T, N, and American Joint Committee on Cancer (AJCC) stages (*p* < 0.01) and significantly correlated to poor progression-free survival (PFS) and overall survival (OS) of 913 ccRCC patients in FUSCC and TCGA cohorts. Pearson's correlation coefficient indicated that *FASN* amplification was positively correlated to VAT% (*r* = 0.772, *p* < 0.001), which significantly correlated to poor PFS (HR = 2.066, *p* = 0.028) and OS (HR = 2.773, *p* = 0.023) in the FUSCC cohort. Transient inhibition or overexpression of *FASN* significantly regulated A498 and 786O cell proliferation and migration by regulating epithelial–mesenchymal transition. Inhibition of *FASN* led to a higher apoptotic rate and decreased lipid droplet formation compared with normal control in ccRCC cells. Non-targeted metabolomics showed that decreased *de novo* lipogenesis might be required to sustain an elevation of glycolytic activity in 786O cells by regulating galactinol, dl-lactate, N-acetylaspartylglutamate, and sucrose, thereby participating in carcinogenesis and progression of ccRCC.

**Conclusion:** This study demonstrated that *FASN* expression is positively related to aggressive cell proliferation, migration, apoptosis, and lipid droplet formation and regulates metabolic disorders of the ccRCC microenvironment. Additionally, elevated *FASN* mRNA expression is significantly correlated to the abdominal obesity distribution, especially VAT%, which is a significant predictor of a poor prognosis for ccRCC patients.

## Introduction

Renal cell carcinoma (RCC) is a highly malignant tumor that originates from the urinary tubular epithelial system of the renal parenchyma. The incidence of RCC is increasing at a rate of 2% per year, especially in developed countries (Siegel et al., 2019). Pathologically, clear cell renal cell carcinoma (ccRCC) accounts for ~70% of all cases (Srigley et al., 2013). ccRCC is highly aggressive and has a poor prognosis, even in patients diagnosed at an early stage and treated by nephrectomy. Thus, there is an urgent need for accurate biomarkers for clinical guidance and prognosis prediction as well as new drug development for treatment.

Accumulating studies have shown that changes in the lipid profile and abnormal lipid metabolism play a major role in the development of cancers, which may provide a new approach for cancer detection and treatment (Xu et al., 2020). Fatty acid synthase (FASN), one of the important enzymes involved in lipid metabolism, synthesizes long-chain fatty acids and largely controls deposition of animal liposomes. Compared with normal cells, all esterified fatty acids in most tumor cells are synthesized *de novo*, and expression of FASN in tumor cells is significantly increased, which leads to aggressiveness and a poor prognosis (Menendez and Lupu, 2007; Horiguchi et al., 2008; Ogino et al., 2008). Therefore, it is worth exploring the relationship between FASN and the occurrence and development of ccRCC.

Previous studies have indicated that obesity, which is defined as a body mass index (BMI) greater than 30 kg/m^2^, is a risk factor for ccRCC (Motamedinia et al., 2008; Renehan et al., 2008; Choi et al., 2013). Although BMI is an internationally recognized convenient and effective weight indicator, many studies have recently disputed the accuracy of BMI to indicate body fat distribution and metabolic risk (Thomas et al., 2012; Peterson et al., 2014; Tomiyama et al., 2016). Therefore, it is essential to determine a new measurement to indicate the distribution of adipose tissue and its prognostic value for ccRCC and other cancers (Ludescher et al., 2009; Britton et al., 2013).

In 2010, Ibrahim indicated that anatomy and biological functions were greatly distinguished between subcutaneous adipose tissue (SAT) and visceral adipose tissue (VAT) in the heterogeneous nature of obesity (Ibrahim, 2010). A recent study showed that a high Fuhrman grade significantly correlates to increased VAT in ccRCC patients (Zhu et al., 2013). The visceral obesity percentage (VAT%) is calculated using the formula VAT% = [(AP – SAT) / AP] × 100% and particularly associated with the progression and prognosis of ccRCC patients (Xu et al., 2019a). The increasing number of studies on obesity distribution has provided novel insights into signaling pathways involved in the occurrence of ccRCC to prevent its progression.

To define the prognostic implications of *FASN* mRNA expression in ccRCC patients and the correlation between *FASN* expression and adipose tissue distribution, we used 533 ccRCC samples from The Cancer Genome Atlas (TCGA) cohort and 380 patients who had received radical nephrectomy. Functional analyses of *FASN* in human ccRCC cells were performed *in vitro*. Additionally, functional annotations of hub genes with FASN and non-targeted metabolomics were used to reveal significant pathways involved in ccRCC carcinogenesis. We hypothesized that elevated *FASN* mRNA expression correlates with VAT%, a poor prognosis, and malignant biological behaviors of ccRCC.

## Materials and Methods

### Clinical Sample Collection and Variables Baseline

This study screened 523 consecutive patients who underwent radical or partial nephrectomy at the Department of Urology, Fudan University Shanghai Cancer Center (FUSCC, Shanghai, China) with available pathology reports and electronic medical records. Among the 143 excluded patients, 27 patients were diagnosed with benign renal tumor, 38 with urinary tract carcinoma, 49 with non-clear RCC, and 29 failed to pass pathological quality check. A total of 380 ccRCC patients were eventually and consecutively enrolled in analyses from August 2009 to November 2018.

This study also consecutively included 533 ccRCC patients with available RNA sequence data from TCGA database. Tissue samples, including ccRCC and adjacent normal tissue, were collected during surgery and fixed in 4% paraformaldehyde, available from FUSCC tissue bank. Clinicopathological parameters of all patients, including age at surgery, gender, BMI, tumor laterality, TNM stage, American Joint Committee on Cancer (AJCC) stage, and International Society of Urological Pathology (ISUP) grade, were collected. Anthropometric measurements of obesity on magnetic resonance imaging (MRI), including anterior abdominal adipose thickness (A in blue line), posterior abdominal adipose thickness (P in pink line), SAT, and VAT, were also collected and analyzed in this study.

### Total mRNA Extraction and Real-Time Quantitative PCR

Total RNA sequence was isolated using TRIzol reagent (Invitrogen, Carlsbad, CA, USA) from 380 paired ccRCC and adjacent normal samples or cells. SYBR® Premix Ex TaqTM (TaKaRa) was implemented to perform RT-qPCR reactions in triplicate according to attached protocols, as previously described (Xu et al., 2019b). The primers of *FASN* were forward: 5′-CGA CAG CAC CAG CTT CGC CA-3′ and reverse: 5′-CAC GCT GGC CTG CAG CTT CT-3′. The relative *FASN* expression quantity was measured after the 2^−ΔΔCt^ calculation using beta-Actin as the internal standard. Relative expression in this study was represented using the ratio of *FASN* expression in Tumor/Normal tissues (T/N).

### Anthropometric Measurements of Obesity

The quantity of anterior abdominal adipose thickness (A in blue line), posterior abdominal adipose thickness (P in yellow line), and anteroposterior diameter (AP in pink line) was measured by MRI at the umbilical level (~L4–L5 level marked in green dotted line) with T2-weighted sagittal localization images for 380 patients. The visceral obesity percentage was defined as VAT%. The value of SAT and VAT% was calculated using the formula SAT = A + P and VAT% = [(AP – SAT) / AP], respectively.

### Human ccRCC Cell Culture

Two normal types of human ccRCC cell lines (A498 and 786O) were obtained from American Type Culture Collection (ATCC). The A498 and 786O cells were cultured in culture medium RPMI-1640 (GIBCO, USA) and supplemented with 10% fetal bovine serum (Hyclone, Life Sciences, Shanghai, China) and 100 U/ml penicillin (Beyotime, China). The A498 and 786O cells were incubated in a humidified atmosphere incubator of 5% CO_2_ at 37°C temperature.

### Cell Transfection

Both A498 and 786O cells have been transfected with double stranded siRNA according to the manufacturer's protocol using plasmid using Lipofectamine 2000 reagent (RiboBio) in a six-well plate. The transfection dose for each well was 15 μl of siRNA-1 (assay ID: 107315; Invitrogen, Carlsbad, CA, USA), siRNA-2 (assay ID: 107316; Invitrogen, Carlsbad, CA, USA), or negative control RNAi (12935-400; Invitrogen, Carlsbad, CA, USA) at a concentration of 150 nM in 250 μl RPMI-1640 incubated for 20 min. The A498 and 786O cells were harvested for at least 24 h after transfection for further experimental analysis.

### Protein Isolation and Western Blot Analysis

Proteins were extracted from A498 and 786O cells using RIPA lysis buffer (Beyotime Biotechnology Shanghai, China) and concentrated by the bicinchoninic acid protein assay kit (Beyotime Biotechnology, Shanghai, China). Samples were separated by electrophoresis on 6 or 10% SDS gel and then transferred to a methanol activated polyvinylidene fluoride (PVDF) membrane. Membranes were blocked with 5% bovine serum albumin (BSA) for 1 h at room temperature and then incubated with primary antibodies, anti-FASN (1:1,000, ab128870, Abcam), and anti-beta-Actin primary antibody (1:3,000, ab179467, Abcam) at 4°C overnight. After washed with TBST for three times, membranes were incubated with secondary antibody goat anti-rabbit IgG conjugated with HRP (1:3,000, ab205718, Abcam) at room temperature for 60 min. After three washes with TBST for 10 min each, the bands were visualized using ECL-plus™ western blotting chemiluminescence kits (BD Biosciences, NJ, USA).

### Cell Viability Analysis

For viability assays, cells treated with shRNA or inhibitors were seeded onto 96-well plates (2,000 cells/well). Next, 10 μl CCK8 solution (KeyGEN BioTECH, Nanjing, China) was added to each well, and cells were incubated at 37°C for 2 h. The absorbance of each well at 450 nm was measured at 1, 2, 3, 4, and 5 days after seeding using an automatic microplate reader (TEAN, Swiss). Three replicate analyses were performed for each sample.

### Cell Apoptosis Assays

Apoptosis detection assay was performed using Annexin V-FITC Apoptosis Detection Kits (BD, USA) in accordance with the manufacturer's procedures. Briefly, the A498 and 786O cells were obtained, triple washed with PBS, and then added 500 μl in 1 × binding buffer. Then, 500 μl cell suspension, 5 μl Annexin V-FITC, and 5 μl propidium iodide (PI) solution were resuspended in each collection tube. After incubation for 15 min, cell apoptosis was analyzed using a FACS analyzer (BD, USA).

### Transwell Migration Assay

After trypsinized and suspended in the medium, 1 × 10^5^ A498 and 786O cells were seeded in medium with 10% FBS and placed in each transwell chamber. The medium containing 20% fetal bovine serum was added in the lower 24-well plate chamber. After 24 h, the bottom A498 and 786O cells were treated with 4% polyoxymethylene for 15 min, deionized water, and 0.1% crystal violet for 30 min. Finally, the A498 and 786O cells migrating to the lower surface of transwell chamber were counted using a microscope in six random fields.

### Oil Red O Staining of Lipid Droplets

Cells were seeded in 12-well plates and cultured in RPMI-1640 10% FCS for 48 h before discarded and replaced by basal media, ACM40. Next, cells were washed twice with DPBS and fixed with 4% paraformaldehyde. Oil Red O staining was performed using 0.3% Oil Red O staining solution dissolved in isopropanol. Lipid droplets were visualized using bright field microscopy with 20 × magnification (Zeiss Primovert, Shanghai, China).

### Survival Analysis

The primary endpoint was overall survival (OS), which was assessed from the date of receiving radical nephrectomy to the date of death or the last follow-up. Progression-free survival (PFS) was the secondary endpoint and was defined as the length of time from the date of surgery to the date of progression, second-line treatment, or death, whichever occurred first. Survival curves were established using the Kaplan–Meier method and analyzed by log-rank test with 95% confidence intervals (CIs). To find independent predictors, the hazard ratio (HR) estimates and 95% CIs were performed using univariate and multivariate Cox logistic regression models. As a supplement to survival, Cox logistic regression analysis was performed on 117 patients with available MRI scan to assess confounding covariates including A, P, SAT, VAT%, TNM stage, ISUP grade, and FASN expression.

### Protein–Protein Interaction Network Construction

Search Tool for the Retrieval of Interacting Genes (STRING; http://string-db.org) (version 10.0) online database was utilized to detect protein–protein interaction (PPI) network of co-regulated hub genes and analyze the functional interactions between relative proteins. An interaction with specificity scores higher than 0.4 was regarded as statistically significant.

### Functional Annotations Analysis

Database for Annotation, Visualization and Integrated Discovery (DAVID; http://david.ncifcrf.gov; version 6.8) online database was utilized to investigate the Gene Ontology (GO): biological process (BP), GO: molecular function (MF), and Kyoto Encyclopedia of Genes and Genomes (KEGG) pathways analyses and then visualized in a bubble chart. To predict potential hallmarks, gene set enrichment analysis (GSEA) was utilized to test significant genes using transcriptional sequences in TCGA database. A permutation test with 1,000 times was used to identify the significantly changed pathways. The adjusted *p* (adj. *p*) values less than 0.01 and false discovery rate (FDR) less than 0.25 were confirmed as significant related genes.

### Analysis of Non-targeted Metabolomics Analysis Process

This experiment uses UHPLC-Q-TOF MS technology combined with data-dependent acquisition methods to perform full-spectrum analysis of samples. AB Triple TOF 5600/6600 mass spectrometer (AB SCIEX) and Agilent 1290 Infinity LC Ultra High-Pressure Liquid Chromatograph (Agilent) were used to obtain primary and secondary mass spectrometry data at the same time and then used XCMS to perform peak extraction and metabolite identification on the data. Waters, ACQUITY UPLC BEH Amide 1.7 μm, 2.1 mm × 100 mm column and HSS T3 1.8 μm, 2.1 mm × 100 mm column were used as the columns.

### Statistical Analysis

All statistical analyses and graphical plotting were performed with SPSS (version 23.0), GraphPad Prism 8 software, or R software (version 3.3.2). Chi-squared test was utilized to find out the association between different *FASN* mRNA expression sets and categorical clinicopathological data distributions. Pearson's correlation coefficient was utilized to determine the association between VAT% and levels of *FASN* mRNA expression. All hypothetical tests were two-sided, and *p*-values < 0.05 were considered significant in all tests.

## Results

This study was conducted in three phases. First, we assessed significant differential *FASN* mRNA expression and its novel prognostic implications in 913 ccRCC patients of TCGA and FUSCC cohorts. Second, we estimated relationships among the VAT percentage, *FASN* expression, and prognosis of ccRCC patients in the FUSCC cohort. Functional experiments of *FASN* in human ccRCC cells were also performed *in vitro*. Third, functional annotations of hub genes with FASN and non-targeted metabolomics were used to reveal significant pathways involved in ccRCC carcinogenesis.

### Differential *FASN* mRNA Expression and Its Correlation With Advanced Clinicopathological Parameters of ccRCC Patients in TCGA Cohort

**Figure 1:**
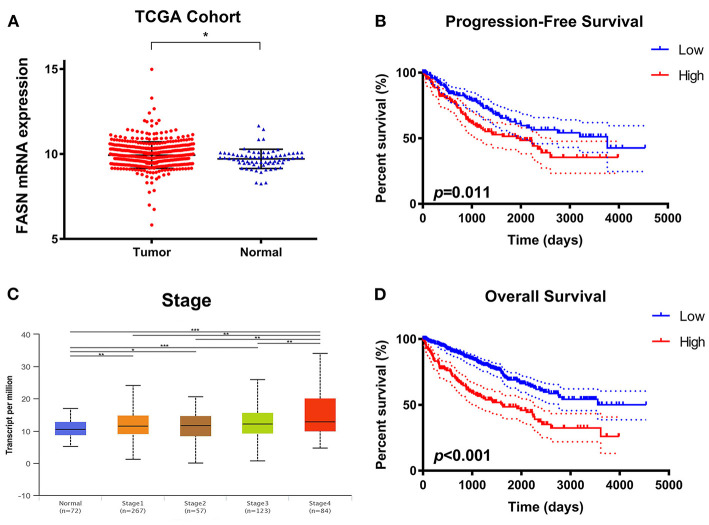
Differential *FASN* mRNA expression and its correlation with advanced clinicopathological parameters in ccRCC patients from TCGA cohort. **(A)** To explore the potential relationship between *FASN* expression and clinicopathological features of ccRCC patients, we enrolled 533 ccRCC samples and 72 adjacent normal samples from TCGA cohort and found significantly increased *FASN* mRNA expression in ccRCC compared with normal samples (*p* < 0.05). **(B)** The *FASN* mRNA expression was found to be markedly related with advanced clinical AJCC stage (*p* < 0.05) and reached the highest in advanced stage 4 **(C,D)** Survival analysis in the Kaplan–Meier method indicated that elevated *FASN* expression was significantly associated with shorter PFS (*p* = 0.011) and OS (*p* < 0.001) in TCGA cohort.

To explore potential relationships between *FASN* expression and clinicopathological features of ccRCC patients, we used 533 ccRCC samples and 72 adjacent normal samples from TCGA cohort and found significantly increased *FASN* mRNA expression in ccRCC samples compared with normal samples (^*^p < 0.05; Figure 1A). Additionally, *FASN* mRNA expression was markedly related to an advanced clinical AJCC stage (*p* < 0.05) and reached its highest in advanced stage 4 (Figure 1B). Kaplan–Meier analysis showed that elevated *FASN* expression was significantly associated with shorter PFS (*p* = 0.011) and OS (*p* < 0.001) in TCGA cohort (Figures 1C,D). Overall, elevated *FASN* mRNA expression was significantly correlated to advanced clinicopathological features and a poor prognosis of 533 ccRCC patients in TCGA cohort.

### Clinicopathological Characteristics of 380 ccRCC Patients in the FUSCC Cohort

We also examined 380 ccRCC patients with paired tumor and normal samples in the FUSCC cohort. Clinicopathological characteristic baselines of 380 patients in relation to the *FASN* expression status are shown in Table 1. Chi-squared tests showed that the characteristic information was balanced in terms of the distribution of categorical data. Increased *FASN* mRNA expression in ccRCC patients was significantly correlated to an advanced T stage (*p* < 0.001), N stage (*p* = 0.019), and AJCC stage (*p* = 0.002) in ccRCC patients of the FUSCC cohort.

**Table 1:** Clinicopathological characteristics in relation to FASN expression status in 380 ccRCC patients from FUSCC cohort.

**Variable**	**Entire group (*n* = 380)**	**FASN mRNA expression**		**χ^2^**	***p*-value**
		**Low expression (*n* = 190)**	**High expression (*n* = 190)**		
Age at surgery (y, median ± SD)		54.1 ± 12.2	56.2 ± 11.4		
BMI (kg/m^2^, median ± SD)		24.5 ± 3.3	23.5 ± 8.3		
Sex (*n*, %)				0.012	0.914
Male	249 (65.5)	125 (65.8)	124 (65.3)		
Female	131 (34.5)	65 (34.2)	66 (34.7)		
Laterality (*n*, %)				0.168	0.682
Left	190 (50.0)	97 (51.1)	93 (48.9)		
Right	190 (50.0)	93 (48.9)	97 (51.1)		
T stage at presentation (*n*, %)				**16.645**	**<0.001**
T1–T2	309 (81.3)	170 (89.5)	139 (73.2)		
T3–T4	71 (18.7)	20 (10.5)	51 (26.8)		
N stage at presentation (*n*, %)				**5.463**	**0.019**
N0	333 (87.6)	174 (91.6)	159 (83.7)		
N1	47 (12.4)	16 (8.4)	31 (16.3)		
M stage at presentation (*n*, %)				2.522	0.112
M0	310 (81.6)	161 (84.7)	149 (78.4)		
M1	70 (18.4)	29 (15.3)	41 (21.6)		
AJCC stage				**9.997**	**0.002**
I–II	292 (76.8)	159 (83.7)	133 (70.0)		
III–IV	88 (23.2)	31 (16.3)	57 (30.0)		
ISUP grade (*n*, %)				0.675	0.411
1–2	182 (47.9)	95 (50.0)	87 (45.8)		
3–4	192 (52.1)	95 (50.0)	103 (54.2)		

*ccRCC, clear cell renal cell carcinoma; FUSCC, Fudan University Shanghai Cancer Center; BMI, body mass index; AJCC, American Joint Committee on Cancer*.

*p value less than 0.05 was considered as statistically significant and marked in bold.

### Validation of Differential *FASN* Expression and Its Prognostic Implication in ccRCC Tissues From the FUSCC Cohort

**Figure 2:**
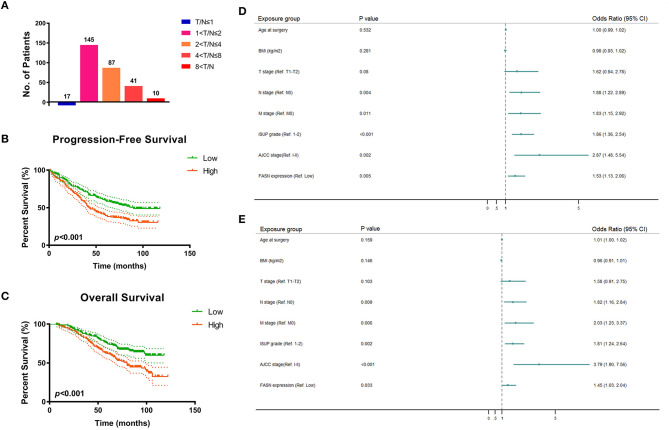
FASN mRNA expression and prognostic implication for 380 ccRCC patients from FUSCC cohort. **(A)** In order to validate *FASN* mRNA expression levels in ccRCC tissues *in vitro*, we performed real-time qPCR on 380 pairs of ccRCC and normal samples from FUSCC cohort. The result showed that the ratio of T/N was dramatically different among the distinct *FASN* mRNA expression group (5.6% in T/N ≤ 1, 48.3% in 1 < T/N ≤ 2, 29% in 2 < T/N ≤ 4, 13% in 4 < T/N ≤ 8, 2.6% in 8 < T/N). **(B,C)** Survival analysis demonstrated that patients with higher *FASN* mRNA expression exhibited significantly poor PFS (*p* < 0.001) and OS (*p* < 0.001). For relative low *FASN* expression patients, the median PFS was 65 months, and the median OS was 71.5 months, respectively. For relative high *FASN* expression patients, the median PFS was 41 months, and the median OS was 65 months. **(D,E)** Forest plot showed multivariate Cox regression analyses that pN stage, pM stage, ISUP grade, and AJCC stage were still relevant to both PFS (pN stage: *p* = 0.004, pM stage: *p* = 0.011, ISUP grade: *p* **<** 0.001, AJCC stage: *p* = 0.002) and OS (pN stage: *p* = 0.009, pM stage: *p* = 0.006, ISUP grade: *p* = 0.002, AJCC stage: *p* **<** 0.001). Importantly, elevated *FASN* expression was significantly correlated with poor PFS (HR = 1.529, *p* = 0.005) and OS (HR = 1.450, *p* = 0.033) in 380 ccRCC patients from FUSCC cohort.

To validate *FASN* expression in ccRCC tissues *in vitro*, we performed real-time quantitative PCR (RT-qPCR) analysis of 380 pairs of ccRCC and normal samples from the FUSCC cohort. The results showed that the ratio of T/N was dramatically different among the distinct *FASN* mRNA expression groups (5.6% in T/N ≤ 1, 48.3% in 1 < T/N ≤ 2, 29% in 2 < T/N ≤ 4, 13% in 4 < T/N ≤ 8, and 2.6% in 8 < T/N; Figure 2A). Survival analysis demonstrated that patients with high *FASN* mRNA expression exhibited significantly poor PFS (*p* < 0.001; Figure 2B) and OS (*p* < 0.001; Figure 2C). For relatively low *FASN* expression, the median PFS was 65 months, and the median OS was 71.5 months. For relatively high *FASN* expression, the median PFS was 41 months, and the median OS was 65 months.

### Univariate and Multivariate Cox Regression Analyses of the FUSCC Cohort

Univariate and multivariate Cox regression analyses were performed for 380 ccRCC patients in the FUSCC cohort. In univariate Cox regression analysis (Supplementary Table 1), traditional prognostic predictors, such as the TNM stage, ISUP grade, and AJCC stage, were markedly correlated to PFS (*p* **<** 0.001) and OS (*p* **<** 0.001) of ccRCC patients. Additionally, age at surgery and BMI were significantly related to PFS (*p* = 0.024, *p* = 0.017) and OS (*p* = 0.021, *p* = 0.012). Importantly, elevated *FASN* expression was significantly associated with poor PFS (HR = 1.854, *p* **<** 0.001) and OS (HR = 2.017, *p* **<** 0.001) of the 380 ccRCC patients in the FUSCC cohort.

In multivariate Cox regression analysis, pN stage, pM stage, ISUP grade, and AJCC stage were still relevant to both PFS (pN stage: *p* = 0.004; pM stage: *p* = 0.011; ISUP grade: *p* **<** 0.001; AJCC stage: *p* = 0.002) and OS (pN stage: *p* = 0.009; pM stage: *p* = 0.006; ISUP grade: *p* = 0.002; AJCC stage: *p* **<** 0.001). Importantly, elevated *FASN* expression was significantly correlated to poor PFS (HR = 1.529, *p* = 0.005) and OS (HR = 1.450, *p* = 0.033) of patients in the FUSCC cohort as displayed in forest plots (Figures 2D,E).

### VAT% Is a More Accurate Obesity Indicator for ccRCC Patients in the FUSCC Cohort

**Figure 3:**
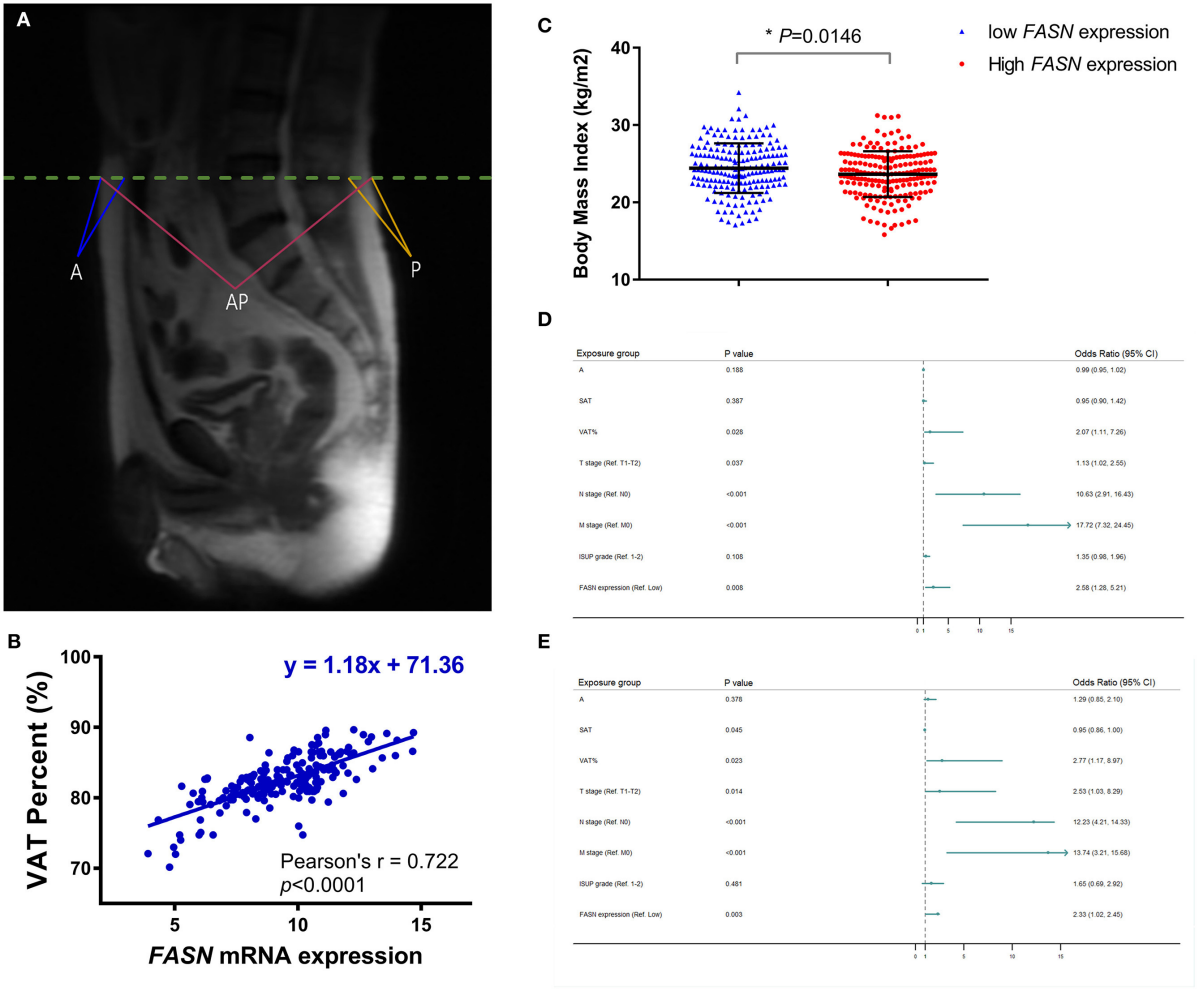
Anthropometric measurement of abdominal adipose and prognostic implication of VAT%. **(A)** The quantity of anthropometric measurements of SAT area was measured by MRI with T2-weighted sagittal localization images. **(B)** Pearson's correlation coefficient demonstrated that elevated FASN mRNA expression was positively correlated with the VAT% (*r* = 0.722, *p* < 0.0001). **(C)** BMI in ccRCC patients with low *FASN* expression is higher than that in patients with high expression of *FASN* (*p* = 0.0146). **(D,E)** In multivariate Cox regression analyses of PFS and OS in 117 ccRCC cases whose MRI scans were available from FUSCC cohort, high VAT% was significantly associated with poor PFS [HR = 2.066, *p* = 0.028; **(D)**] and OS [HR = 2.773, *p* = 0.023; **(E)**]. However, BMI was not an independent covariate affecting survival. In addition, advanced pT (HR = 1.132, *p* = 0.037), pN (HR = 10.63, *p* **<** 0.001), pM (HR = 17.72, *p* **<** 0.001), and *FASN* expression (HR = 2.578, *p* = 0.008) was significantly correlated with poorer PFS. SAT (HR = 0.955, *p* = 0.045), advanced pT (HR = 2.526, *p* = 0.014), pN (HR = 12.23, *p* **<** 0.001), pM (HR = 13.736, *p* **<** 0.001), and FASN expression (HR = 2.33, *p* = 0.003) was also markedly related with poor OS in 117 ccRCC cases.

To confirm that VAT% is a better prognostic predictor than BMI (Figure 3A) based on MRI scanning, we assessed correlations between VAT, BMI, and *FASN* mRNA expression. Pearson's correlation coefficient suggested that elevated *FASN* mRNA expression was positively correlated to VAT% (y = 1.18x + 71.36, *r* = 0.722, *p* **<** 0.0001; Figure 3B). Additionally, we assessed BMI in ccRCC patients of the FUSCC cohort. The results showed that the BMI of patients with low *FASN* expression was significantly higher than that of patients with high *FASN* expression (*p* = 0.0146; Figure 3C).

Univariate Cox analysis indicated that SAT, VAT%, TNM stage, ISUP grade, and *FASN* expression were independent parameters for prognosis of 117 ccRCC patients (Supplementary Table 2). In multivariate Cox regression analyses of PFS and OS in 117 ccRCC cases whose MRI scans were available from the FUSCC cohort, a high VAT% was significantly associated with poor PFS (HR = 2.066, *p* = 0.028; Figure 3D) and OS (HR = 2.773, *p* = 0.023; Figure 3E). However, BMI was not an independent covariate that affected survival. Additionally, advanced pT (HR = 1.132, *p* = 0.037), pN (HR = 10.63, *p* **<** 0.001), pM (HR = 17.72, *p* **<** 0.001), and *FASN* expression (HR = 2.578, *p* = 0.008) were significantly correlated to poorer PFS (Figure 3D). SAT (HR = 0.955, *p* = 0.045), advanced pT (HR = 2.526, *p* = 0.014), pN (HR = 12.23, *p* **<** 0.001), pM (HR = 13.736, *p* **<** 0.001), and FASN expression (HR = 2.33, *p* = 0.003) were also significantly related to poor OS in 117 ccRCC cases (Figure 3E).

### *FASN* Regulates Proliferation of ccRCC Cells

**Figure 4:**
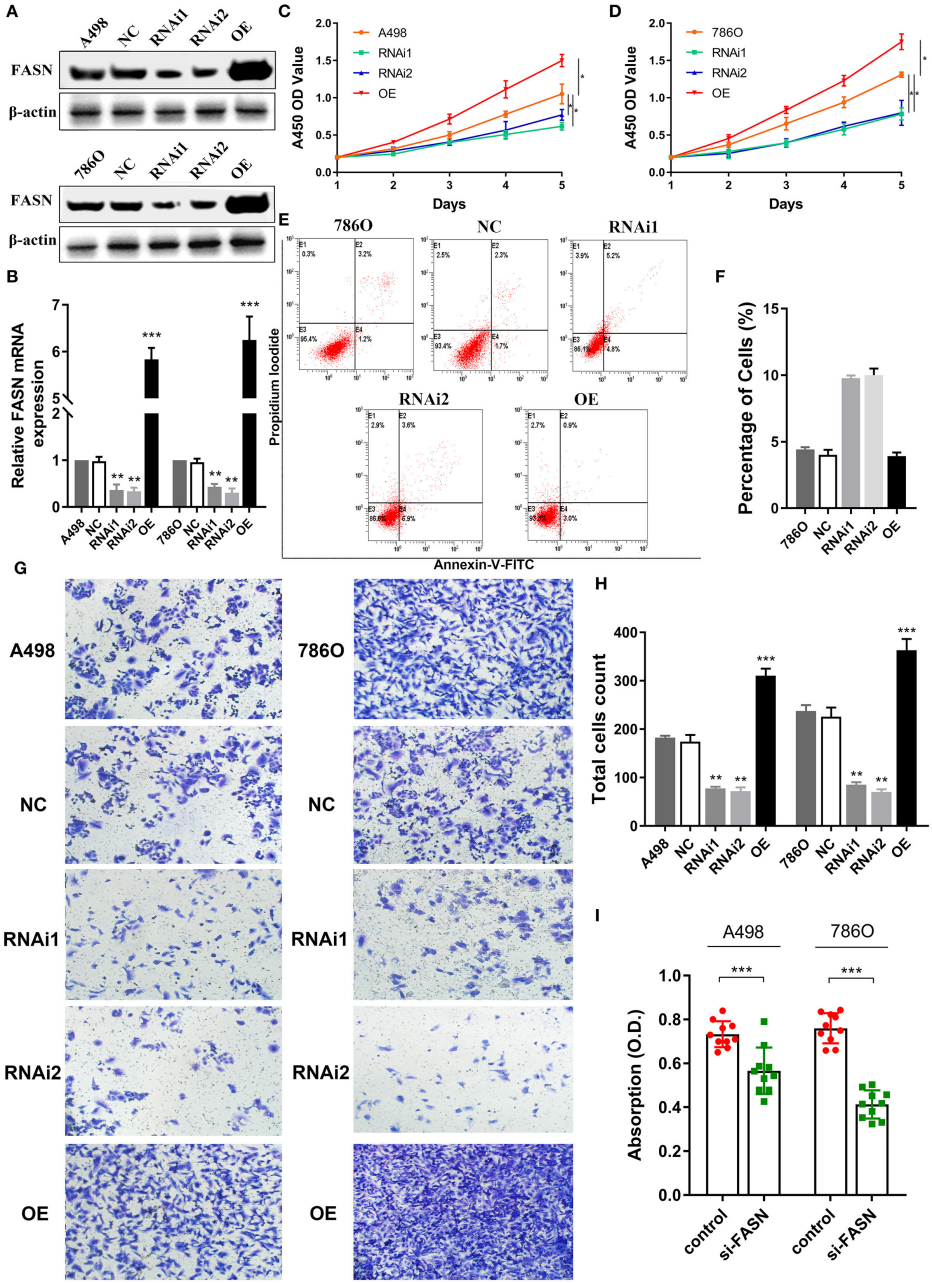
FASN regulates proliferation, migration, apoptosis, and lipid droplets formation of human ccRCC cells. **(A,B)** After transient transfected with FASN plasmid in normal control, *FASN*-RNAi1, *FASN*-RNAi2, and *FASN* overexpression (OE) groups, we investigated *FASN* mRNA and protein expression level in A498 and 786O cells and found significant differential expression levels in the RNAi1, RNAi2, and OE groups compared with A498 and 786O cells. **(C,D)** After the A498 and 786O cells were transfected for 5 days, the A450 OD values revealed that cells proliferation ability was significantly suppressed in the RNAi1 and RNAi2 groups and significantly promoted in the OE groups compared with normal control (*p* < 0.05). **(E,F)** The X and Y axes represent Annexin V-FITC and PI, respectively. Apoptotic cells number was increased in the RNAi1- and RNAi2-transfected groups compared with control in 786O cells, whereas the *FASN* OE groups showed similar apoptosis to normal control cells. **(G,H)** To explore the role of FASN-mediated migration ability of ccRCC cells, we performed transwell migration assay and found that the inhibition of *FASN* markedly restrained migrated cell numbers. Meanwhile, the OE of *FASN* significantly promotes migrated A498 and 786O cell counts. **(I)** To explore whether FASN regulates ccRCC cells lipid formations, we performed Oil Red O staining and found that inhibition of FASN significantly reduced absorption of lipid droplets in lipid-rich cells. *p < 0.05; **p < 0.01; ***p < 0.001; ****p < 0.0001.

Metabolism reprogramming is a hallmark of cancer and crucial for the progression of ccRCC. We investigated *FASN* expression levels in A498 and 786O cells after transfection with normal control, *FASN*-RNAi1, *FASN*-RNAi2, and *FASN* overexpression (OE) plasmids in addition to cell viability and apoptosis. *FASN* mRNA and protein expression was significantly decreased in *FASN*-RNAi-transfected cells, but increased in the *FASN* OE-transfected groups of A498 and 786O cells (*p* < 0.05, Figures 4A,B, Supplementary Figure 1A). After the A498 and 786O cells were transfected for 5 days, CCK8 assays revealed that cell proliferation was significantly suppressed in the RNAi1- and RNAi2-transfected groups and significantly promoted in the OE groups compared with normal controls (*p* < 0.05) (Figures 4C,D).

### Inhibition of *FASN* May Increase Apoptosis of 786O Cells

As shown in Figure 4E, we detected apoptosis in A498 and 786O cells of the NC, RNAi1, RNAi2, and OE groups. Annexin V-FITC (–) PI (–), E3, stands for normal cells; Annexin V-FITC (+) PI (–), E4, stands for early apoptotic cells; Annexin V-FITC (+) PI (+), E2, stands for late apoptotic cells; Annexin V-FITC (–) PI (+), E1, stands for mechanical necrotic cells. Apoptotic cells number was increased in the RNAi1- and RNAi2-transfected groups compared with control in 786O cells, whereas the *FASN* OE groups showed similar apoptosis to normal control cells (Figure 4F). Apoptotic cells number was increased in the RNAi1-transfected groups compared with control in A498 cells, whereas the *FASN* OE groups showed similar apoptosis to normal control (Supplementary Figures 1B,C).

### *FASN* Regulates Cell Migration and Lipid Droplet Formation

To explore FASN-mediated migration of ccRCC cells, we performed transwell migration assays and found that inhibition of *FASN* markedly decreased migrated cell numbers (*p* **<** 0.05). Moreover, OE of *FASN* significantly increased migrated A498 and 786O cell numbers (Figures 4G,H). To explore whether FASN regulated lipid formation in ccRCC cells, we performed Oil Red O staining and found that inhibition of FASN significantly reduced absorption of lipid droplets in lipid-rich ccRCC cells (*p* **<** 0.001; Figure 4I).

### Validation by IHC and Potential Signaling Pathways *in vitro*

**Figure 5:**
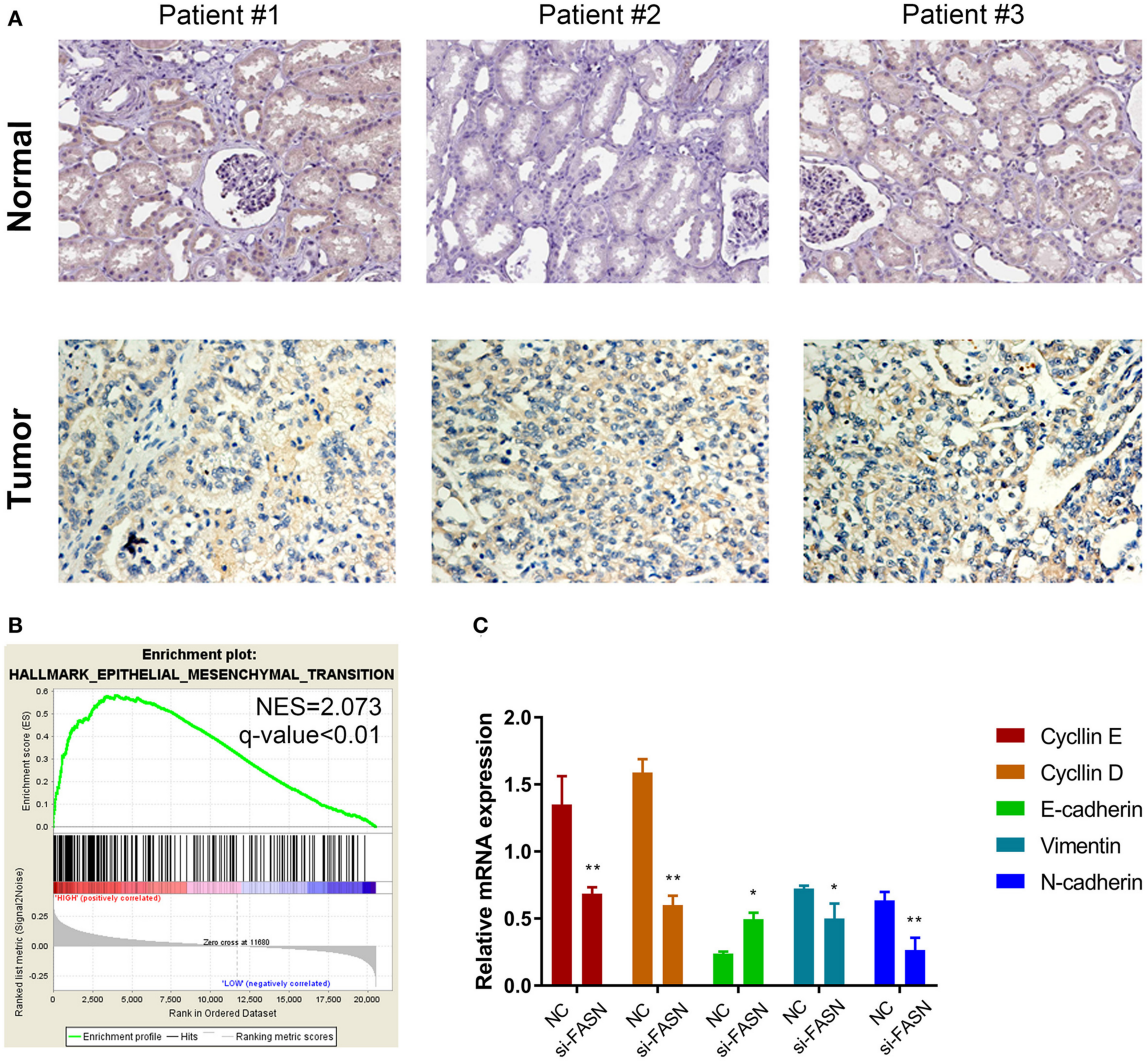
Proteomic validation of FASN and potential signal pathway (especially the EMT process) *in vitro*. **(A)** IHC staining was performed to validate differential expression levels between tumor and normal samples, suggesting a significantly elevated FASN staining density and intensity in ccRCC tissues compared with normal tissues. **(B)** GSEA also suggested that *FASN*, together with related hub genes, was significantly involved in epithelial–mesenchymal transition process (NES = 2.073), consistent with the role of *FASN*-mediated ccRCC cells migration ability. **(C)** RT-qPCR reveals differential mRNA expression of hub genes in epithelial–mesenchymal transformation process, including cyclin E, cyclin D, E-cadherin, vimentin, and N-cadherin. The finding indicated that inhibition of *FASN* significantly decreased cyclin E, cyclin D, vimentin, and N-cadherin expression (*p* < 0.05) and increased E-cadherin mRNA expression (*p* < 0.01). *p < 0.05; **p < 0.01; ***p < 0.001; ****p < 0.0001.

Immunohistochemistry (IHC) staining was performed to validate differential expression levels between tumor and normal samples, which suggested a significantly elevated FASN staining density and intensity in ccRCC tissues compared with those in normal tissues (Figure 5A). Interestingly, GSEA also suggested that *FASN*, together with related hub genes, was significantly involved in epithelial–mesenchymal transition (EMT, NES = 2.073), which was consistent with *FASN*-mediated ccRCC cell migration (Figure 5B).

### *FASN* Promotes Cell Migration by Regulating EMT

RT-qPCR revealed differential mRNA expression of hub genes in EMT, including cyclin E, cyclin D, E-cadherin, vimentin, and N-cadherin. The results indicated that inhibition of *FASN* significantly decreased cyclin E, cyclin D, vimentin, and N-cadherin expression (*p* < 0.05) and increased E-cadherin mRNA expression (*p* < 0.01; Figure 5C).

### Functional Annotations and Predicted Signaling Pathways *in silico*

**Figure 6:**
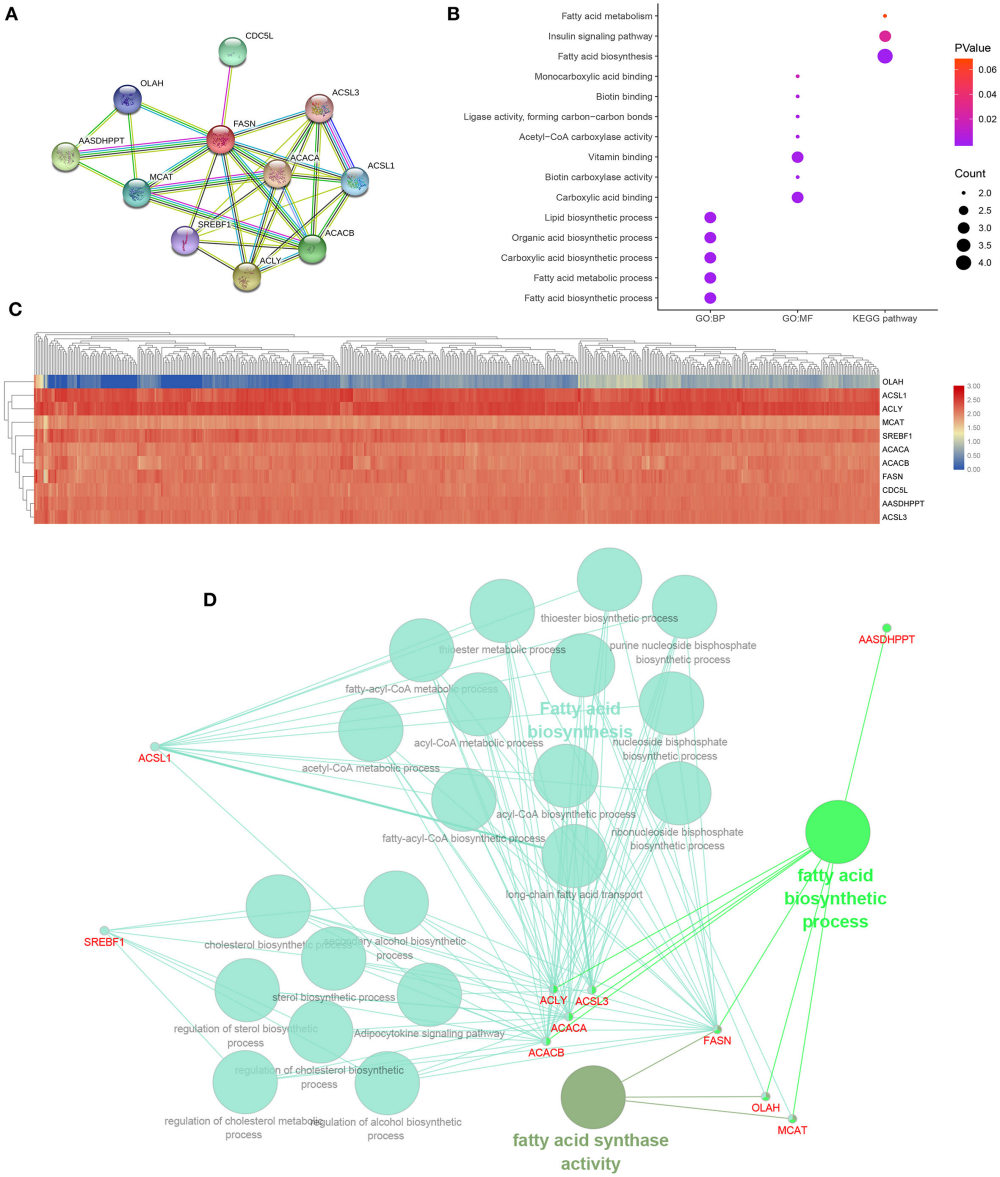
Functional annotations and predicted signaling pathways *in silico*. **(A)** A PPI network of FASN and its co-expression genes was established, including ACACA, ACACB, ACLY, AASDHPPT, ACSL1, ACSL3, CDC5L, MCAT, OLAH, and SREBF1. **(B)** Functional enrichment analyses of a total of 11 involved genes were performed and visualized in a bubble chart. Significant genes involved in fatty acid biosynthetic process, fatty acid metabolic process, carboxylic acid, organic acid, and lipid biosynthetic process markedly participated in carboxylic acid binding and vitamin binding. **(C)** Horizontal and vertical cluster analyses were performed in 533 ccRCC samples from TCGA database and 11 related genes, and the heat map was obtained after standardization. **(D)** Functional annotation using ClueGO indicated that changes in the biological processes of the FASN were significantly associated with fatty acid biosynthesis, fatty acid biosynthetic process, and fatty acid synthase activity.

A PPI network of FASN and its coexpression genes was established, including ACACA, ACACB, ACLY, AASDHPPT, ACSL1, ACSL3, CDC5L, MCAT, OLAH, and SREBF1 (Figure 6A). As shown in Figure 6B, a bubble chart illustrated functional enrichment of 11 related genes. Significant genes involved in fatty acid biosynthetic process, fatty acid metabolic process, carboxylic acid, organic acid, and lipid biosynthetic process markedly participated in carboxylic acid binding and vitamin binding. After normalization of the 11-hub gene transcriptional data, clustering analysis was performed, and a heat map was constructed (Figure 6C). Functional annotations using ClueGO showed that changes in *FASN* biological processes were closely related to fatty acid biosynthesis, fatty acid biosynthetic process, and FASN activity (Figure 6D).

### Significant Genes and Pathways Predicted by GSEA

**Figure 7:**
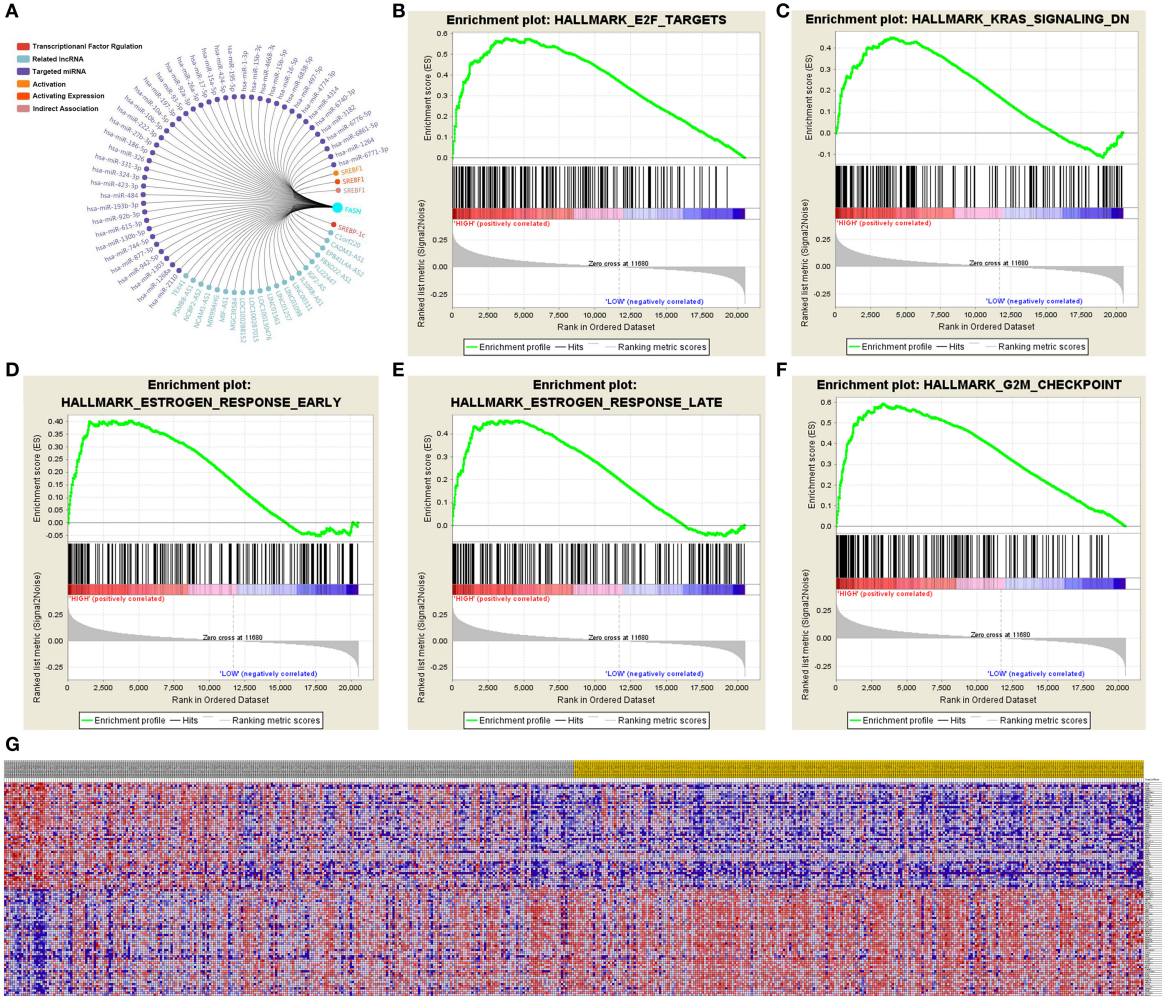
Significant related genes and hallmarks pathways in ccRCC predicted by GSEA. **(A–F)** The most significant pathways include E2F targets, down-regulation of Kras signaling, estrogen response, and G2M checkpoint. **(G)** Transcriptional expression profiles of a total of 100 significant genes with positive and negative correlation were performed in a heat map.

A total of 100 significant genes were obtained from GSEA, and genes with positive and negative correlations were plotted. The results illustrated that the most significant pathways included E2F targets, down-regulation of Kras signaling, the estrogen response, and G2M checkpoint (Figures 7A–F). Additionally, a heat map showed the transcriptional expression profiles of the 100 most significantly up- or down-regulated genes (Figure 7G).

### Non-targeted Metabolomics Reveals Significant Metabolites and Related Pathways of FASN in 786O Cells

**Figure 8:**
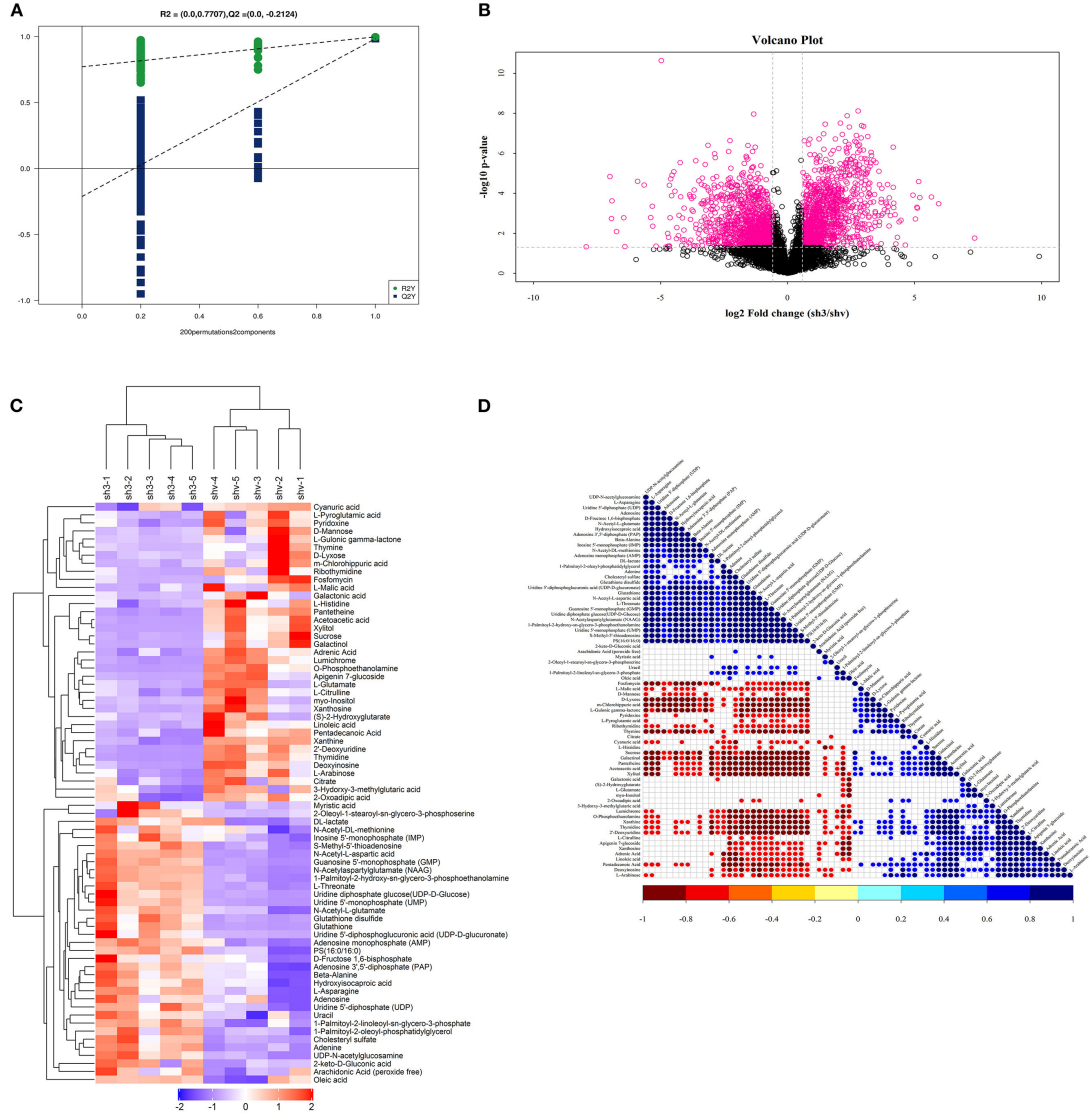
Non-targeted metabolomics reveals significant metabolites and related pathways FASN involved in 786O cells. **(A)** PLS-DA-Permutation analysis suggested marked opposition of the two sets of samples after inhibition of FASN expression in 786O cells. **(B)** Volcano plot suggested significant metabolites with *p* value < 0.05 and log_2_|Fold Change| higher than 1. **(C)** Non-targeted metabolomics found that decreased *de novo* lipogenesis might be required to sustain elevations in glycolytic activity in 786O cells *via* regulating galactinol, dl-lactate, N-acetylaspartylglutamate (NAAG), and sucrose or other significantly involved metabolic pathways, such as pantetheine and adrenic acid. **(D)** Correlation analysis between all significantly involved pathways.

PLS-DA-Permutation analysis suggested marked opposition of the two sets of samples after inhibition of FASN expression in 786O cells (Figure 8A). A volcano plot suggested significant metabolites with a *p*-value of less than 0.05 and log_2_|Fold Change| higher than 1 (Figure 8B). Non-targeted metabolomics found that decreased *de novo* lipogenesis might be required to sustain elevations in glycolytic activity in 786O cells *via* regulating galactinol, dl-lactate, N-acetylaspartylglutamate (NAAG), and sucrose or other significantly involved metabolic pathways, such as pantetheine and adrenic acid (Figure 8C). Correlation analysis between all significantly involved pathways is shown in Figure 8D.

## Discussion

In this study, we investigated whether *FASN* mRNA expression has potential implications in ccRCC patients and its association with the abdominal adipose distribution. To clarify differential *FASN* mRNA expression in ccRCC, we examined differential *FASN* expression between tumor and normal paracancerous tissues and identified prognostic value in 913 ccRCC patients of TCGA and FUSCC cohorts. Next, we assessed anthropometric measurements of adipose distribution based on MRI, which suggested that *FASN* mRNA expression was significantly associated with an elevated VAT%. Importantly, ccRCC patients with up-regulated *FASN* mRNA expression had significantly poor PFS and OS. Additionally, after transfection efficiency was verified, we found that transient inhibition or OE of *FASN* significantly regulated A498 and 786O cell proliferation and migration by regulating EMT. Inhibition of *FASN* resulted in a high apoptotic rate and decreased lipid droplet formation in ccRCC cells.

The correlation between adipose-related genes and tumors has raised new concerns in terms of tumor microenvironment. Several previous studies have elaborated the role of lipid metabolism in tumorigenesis (Calle et al., 2003). Mechanically, obesity leads to overactivation of macrophages in adipose tissue and up-regulation of carcinogenic compounds by changing the signal transmission between adipocytes and other cells (Wright and Simone, 2016). More importantly, *FASN* is involved in the process of lipid metabolism and highly expressed in tumor cells. Several studies have suggested that FASN-mediated *de novo* synthesis of lipids may be a reasonable therapeutic target for the treatment of cancer and adiposity (Bouchi et al., 2015; Higuchi et al., 2017).

Compared with the traditional predictor BMI, this study demonstrated that VAT% is a more accurate risk indicator of obesity related to ccRCC. Although BMI has been accepted to be a risk factor of obesity, it has been challenged because of its rough estimation, including region, ethnic diversity, and failing to distinguish fat distribution (Wang et al., 2014; Xu et al., 2019a). MRI was used to measure SAT as a preoperative examination for RCC patients, and then VAT% was calculated. Although computed tomography is considered to be the standard imaging method to measure abdominal obesity, MRI has advantages of high accuracy and safety (Klopfenstein et al., 2012; Poonawalla et al., 2013; Schaudinn et al., 2015). The thickness of abdominal obesity varies at different levels and should be measured at the level of the umbilicus to reduce the deviation caused by the measurement method.

Non-targeted, high throughput metabolomic profiling deeply analyzes changes of metabolites in the tumor microenvironment, which guide the process of tumor progression and metabotypes (Dubuis et al., 2017). In this study, non-targeted metabolomics showed that decreased *de novo* lipogenesis might be required to sustain elevations in glycolytic activity of 786O cells by regulating galactinol, dl-lactate, NAAG, and sucrose, thereby participating in carcinogenesis and progression of ccRCC. Therefore, in-depth research on the pathological mechanism of FASN may facilitate the analysis of the temporal and spatial dynamics of metabolites and metabolic networks (Dumas et al., 2016; Vantaku et al., 2019).

The advantage of our study is that we first assessed the expression level of FASN mRNA in ccRCC tumor and para-cancerous tissues in two cohorts. Additionally, we found that ccRCC patients with elevated *FASN* mRNA expression had poor PFS and OS, which were consistent with a previous study (Horiguchi et al., 2008). Next, we investigated the correlation between *FASN* expression and the abdominal obesity distribution, especially VAT% that serves as a more representative indicator than BMI to investigate individual adipose distribution of ccRCC patients. Our results showed that expression of *FASN* mRNA was significantly associated with the abdominal adipose distribution and especially positively correlated to increased VAT%. Additionally, we performed functional annotations of *FASN* in ccRCC and its associated signal hallmarks *in vitro* and *in silico*.

However, this study had several limitations. First, our study did not clarify the underlying mechanism of FASN in cellular lipid metabolism of ccRCC. Second, the abdominal obesity distribution was measured by MRI, and VAT% was directly calculated in this study. This measurement method may cause some bias and incur additional costs for patients, whereas only 117 patients had undergone MRI scans. Another limitation in this study is that generalizability and population variety may not be conclusive enough to firmly support our results based on 913 ccRCC patients in FUSCC and TCGA cohorts.

## Conclusion

This study demonstrated that *FASN* expression is positively related to aggressive cell proliferation, migration, apoptosis, and lipid droplet formation and regulates metabolic disorders of the ccRCC microenvironment. Additionally, we revealed that elevated *FASN* mRNA expression was significantly correlated to abdominal obesity distribution, especially VAT%, which is also a significant predictor of a poor prognosis for ccRCC patients.

## Data Availability Statement

The raw data supporting the conclusions of this article will be made available by the authors, without undue reservation.

## Ethics Statement

The studies involving human participants were reviewed and approved by Fudan University Shanghai Cancer Center. The patients/participants provided their written informed consent to participate in this study. Written informed consent was obtained from the individual(s) for the publication of any potentially identifiable images or data included in this article.

## Author Contributions

WX, AA, WL, and XT: conceptualization and validation. WX, AA, XT, JW, and WZ: data curation. WX, AA, XH, WL, JW, and WZ: formal analysis and investigation. Y-YQ, HZ, and DY: funding acquisition and writing—review and editing. WX, AA, XH, JW, and WZ: methodology. FW, XH, and GS: project administration and resources. XT, FW, and GS: software. FW, XH, GS, Y-YQ, HZ, and DY: supervision. WX, AA, XH, WL, and XT: visualization. WX, AA, and XH: roles/writing—original draft. All authors contributed to the article and approved the submitted version.

## Conflict of Interest

The authors declare that the research was conducted in the absence of any commercial or financial relationships that could be construed as a potential conflict of interest.

## Abbreviations

PFS: progression-free survival
OS: overall survival
FUSCC: Fudan University Shanghai Cancer Center
SAT: subcutaneous adipose tissue
VAT: visceral adipose tissue
HR: hazard ratio
CI: confidence interval
MRI: magnetic resonance imaging
BMI: body mass index
ISUP: International Society of Urological Pathology
AJCC: American Joint Committee on Cancer
PPI: protein–protein interaction
GSEA: gene set enrichment analysis
IHC: immunohistochemistry.
