# Supplementary material for: Fatty Acid Synthase Correlates With Prognosis-Related Abdominal Adipose Distribution and Metabolic Disorders of Clear Cell Renal Cell Carcinoma
Source: Front Mol Biosci. 2021 Jan 25;7:610229. doi: 10.3389/fmolb.2020.610229 (PMC7868388; doi:10.3389/fmolb.2020.610229)
Supplement: Supplementary file 1 [file Table_1.DOCX]

**Table S1**. Univariate Cox regression analyses of PFS and OS in 380 enrolled ccRCC patients from FUSCC cohort.

|  | PFS | |  | OS | |
| --- | --- | --- | --- | --- | --- |
| Covariates | HR (95%CI) | *P* value |  | HR (95%CI) | *P* value |
| Age at surgery | **1.013 (1.002-1.025)** | **0.024** |  | **1.015 (1.002-1.028)** | **0.021** |
| BMI (kg/m^2^) | **0.947 (0.906-0.990)** | **0.017** |  | **0.938 (0.892-0.986)** | **0.012** |
| Sex (Ref. Male) | 0.838 (0.626-1.122) | 0.235 |  | 1.000 (0.724-1.381) | 0.999 |
| Laterality (Ref. left) | 1.018 (0.776-1.335) | 0.898 |  | 0.967 (0.710-1.316) | 0.829 |
| T stage (T1-T2) | **7.274 (5.300-9.983)** | **<0.001** |  | **9.198 (6.541-12.933)** | **<0.001** |
| N stage (Ref. N0) | **7.636 (5.341-10.917)** | **<0.001** |  | **8.806 (5.991-12.944)** | **<0.001** |
| M stage (Ref. M0) | **7.842 (5.730-10.734)** | **<0.001** |  | **10.118 (7.171-14.275)** | **<0.001** |
| ISUP grade (Ref. 1-2) | **2.694 (2.019-3.594)** | **<0.001** |  | **3.267 (2.313-4.614)** | **<0.001** |
| AJCC stage(Ref. I-II) | **9.700 (7.066-13.316)** | **<0.001** |  | **13.087 (9.155-18.708)** | **<0.001** |
| FASN expression (Ref. Low) | **1.854 (1.404-2.446)** | **<0.001** |  | **2.017 (1.464-2.778)** | **<0.001** |

Abbreviations: PFS: progression free survival; OS: overall survival; ccRCC: clear cell renal cell carcinoma; FUSCC, Fudan University Shanghai Cancer Center; HR: hazard ratio; CI: confidence interval; BMI: body mass index; ISUP: International Society of Urological Pathology

**P* value less than 0.05 was considered as statistically significance, and marked in bold.

**Table S2**. Univariate Cox regression analyses of PFS and OS in 117 ccRCC cases whose MRI scans were available from FUSCC cohort.

|  | PFS | |  | OS | |
| --- | --- | --- | --- | --- | --- |
| Covariates | HR (95%CI) | *P* value |  | HR (95%CI) | *P* value |
| Age at surgery | 1.022 (0.978-1.094) | 0.289 |  | 1.022 (0.998-1.026) | 0.059 |
| BMI (kg/m^2^) | 0.949 (0.816-1.206) | 0.334 |  | **0.947 (0.911-0.990)** | **0.025** |
| Sex (Ref. Male) | 0.701 (0.394-1.246) | 0.297 |  | 0.875 (0.428-1.889) | 0.781 |
| A | **0.922 (0.829-0.970)** | **0.011** |  | **0.941 (0.865-0.994)** | **0.041** |
| P | 0.968 (0.914-1.057) | 0.233 |  | 0.957 (0.876-1.041) | 0.256 |
| AP | 0.985 (0.979-1.011) | 0.497 |  | 0.944 (0.899-1.011) | 0.097 |
| SAT | **0.923 (0.893-0.977)** | **0.012** |  | **0.966 (0.901-0.993)** | **0.034** |
| VAT% | **3.585 (1.504-11.681)** | **<0.001** |  | **3.267 (2.103-9.682)** | **0.004** |
| Laterality (Ref. left) | 1.046 (0.726-1.519) | 0.843 |  | 1.035 (0.511-2.098) | 0.924 |
| T stage (Ref. T1-T2) | **6.264 (2.003-9.671)** | **<0.001** |  | **9.021 (2.980-11.261)** | **<0.001** |
| N stage (Ref. N0) | **9.146 (4.288-10.395)** | **<0.001** |  | **10.507 (3.481-12.341)** | **<0.001** |
| M stage (Ref. M0) | **11.813 (5.452-17.337)** | **<0.001** |  | **13.971 (5.971-16.541)** | **<0.001** |
| ISUP grade (Ref. 1-2) | **1.728 (1.463-5.614)** | **0.025** |  | **1.876 (1.062-4.081)** | **0.013** |
| *FASN* expression (Ref. Low) | **3.544 (1.892-8.911)** | **<0.001** |  | **2.347 (1.285-2.981)** | **<0.001** |

Abbreviations: PFS, progression-free survival; OS: overall survival; FUSCC, Fudan University Shanghai Cancer Center; MRI, magnetic resonance imaging; BMI, body mass index; SAT, subcutaneous adipose tissue; VAT, visceral adipose tissue; ISUP, International Society of Urological Pathology.

**P* value less than 0.05 was considered as statistically significance, and marked in bold.
