# Supplementary figures and images for: Fatty Acid Synthase Correlates With Prognosis-Related Abdominal Adipose Distribution and Metabolic Disorders of Clear Cell Renal Cell Carcinoma
Source: Front Mol Biosci. 2021 Jan 25;7:610229. doi: 10.3389/fmolb.2020.610229 (PMC7868388; doi:10.3389/fmolb.2020.610229)

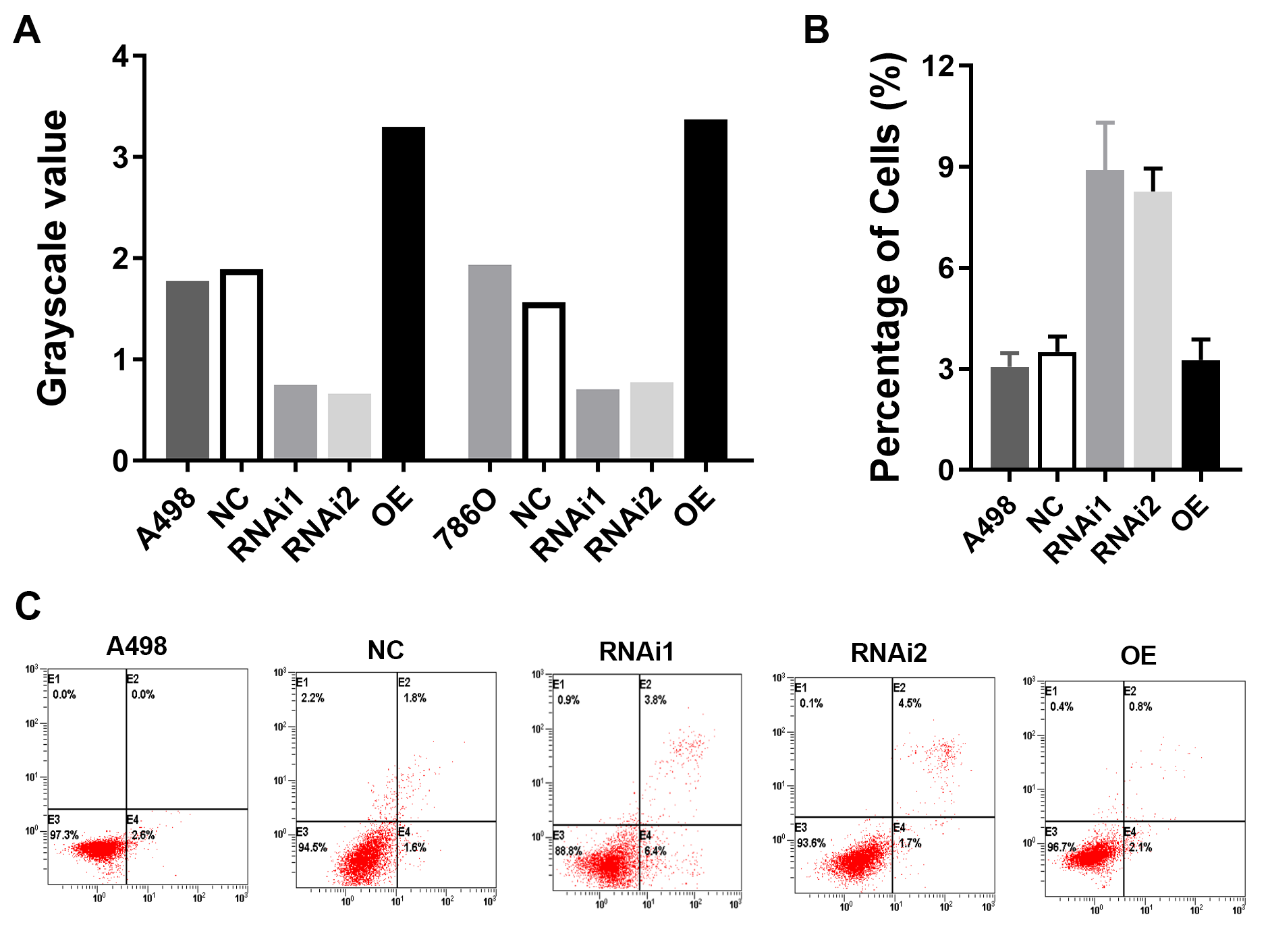

Supplement: Supplementary file 2 [file Image_1.TIF]
